# Summer Laborers

**Published:** 1871-05

**Authors:** 


### SUMMER LABORERS.

Those who have to work in the sun in the heats of summer will find, upon a fair trial, an incalculable advantage by arranging to take their breakfast and be ready to go to work soon after daylight. Leave off work at twelve o'clock. Take dinner, sleep two hours, and begin work again at three; or it might be better to stop at one o'clock and resume work at four, and be in bed at nine o'clock; this will give them full six or seven hours' sleep at night, and nearly two in the daytime. Thus refreshed by a sleep in the afternoon, they will be able to work with the vigor of the morning, and will escape the dreadful task of three hours' labor in the hottest part of the day, which is so fatal to many. Hod-carriers, brick-masons, carpenters, and other mechanics make a sad mistake when they wait until after sunrise of a summer morning before they begin their day's work; the result being, that in the cool of the day they do nothing, and work hard in the boiling sun at noon during the fiercest heats of midsummer. Many of these men may be seen any summer-day, at noon, during the time allowed them for dinner, fast asleep on the bare ground, which, if the least damp, causes them to wake up with a disagreeable tiredness and stiffness, ending after a while in rheumatism, to trouble them through all after life. A coat for a pillow, and even a newspaper spread under them, would be a great protection; but a hard board would be much better, in the absence of a stout coat.

